# New seismological data from the Calabrian arc reveal arc-orthogonal extension across the subduction zone

**DOI:** 10.1038/s41598-020-79719-8

**Published:** 2021-01-12

**Authors:** Tiziana Sgroi, Alina Polonia, Graziella Barberi, Andrea Billi, Luca Gasperini

**Affiliations:** 1 Istituto Nazionale di Geofisica e Vulcanologia, Sezione Roma 2, Roma, Italy; 2 Consiglio Nazionale delle Ricerche, ISMAR, Bologna, Italy; 3 Istituto Nazionale di Geofisica e Vulcanologia (INGV), Osservatorio Etneo, Catania Italy; 4 Consiglio Nazionale delle Ricerche, IGAG, Roma, Italy

**Keywords:** Natural hazards, Solid Earth sciences

## Abstract

The Calabrian Arc subduction-rollback system along the convergent Africa/Eurasia plate boundary is among the most active geological structures in the Mediterranean Sea. However, its seismogenic behaviour is largely unknown, mostly due to the lack of seismological observations. We studied low-to-moderate magnitude earthquakes recorded by the seismic network onshore, integrated by data from a seafloor observatory (NEMO-SN1), to compute a lithospheric velocity model for the western Ionian Sea, and relocate seismic events along major tectonic structures. Spatial changes in the depth distribution of earthquakes highlight a major lithospheric boundary constituted by the Ionian Fault, which separates two sectors where thickness of the seismogenic layer varies over 40 km. This regional tectonic boundary represents the eastern limit of a domain characterized by thinner lithosphere, arc-orthogonal extension, and transtensional tectonic deformation. Occurrence of a few thrust-type earthquakes in the accretionary wedge may suggest a locked subduction interface in a complex tectonic setting, which involves the interplay between arc-orthogonal extension and plate convergence. We finally note that distribution of earthquakes and associated extensional deformation in the Messina Straits region could be explained by right-lateral displacement along the Ionian Fault. This observation could shed new light on proposed mechanisms for the 1908 Messina earthquake.

## Introduction

**Figure 1:**
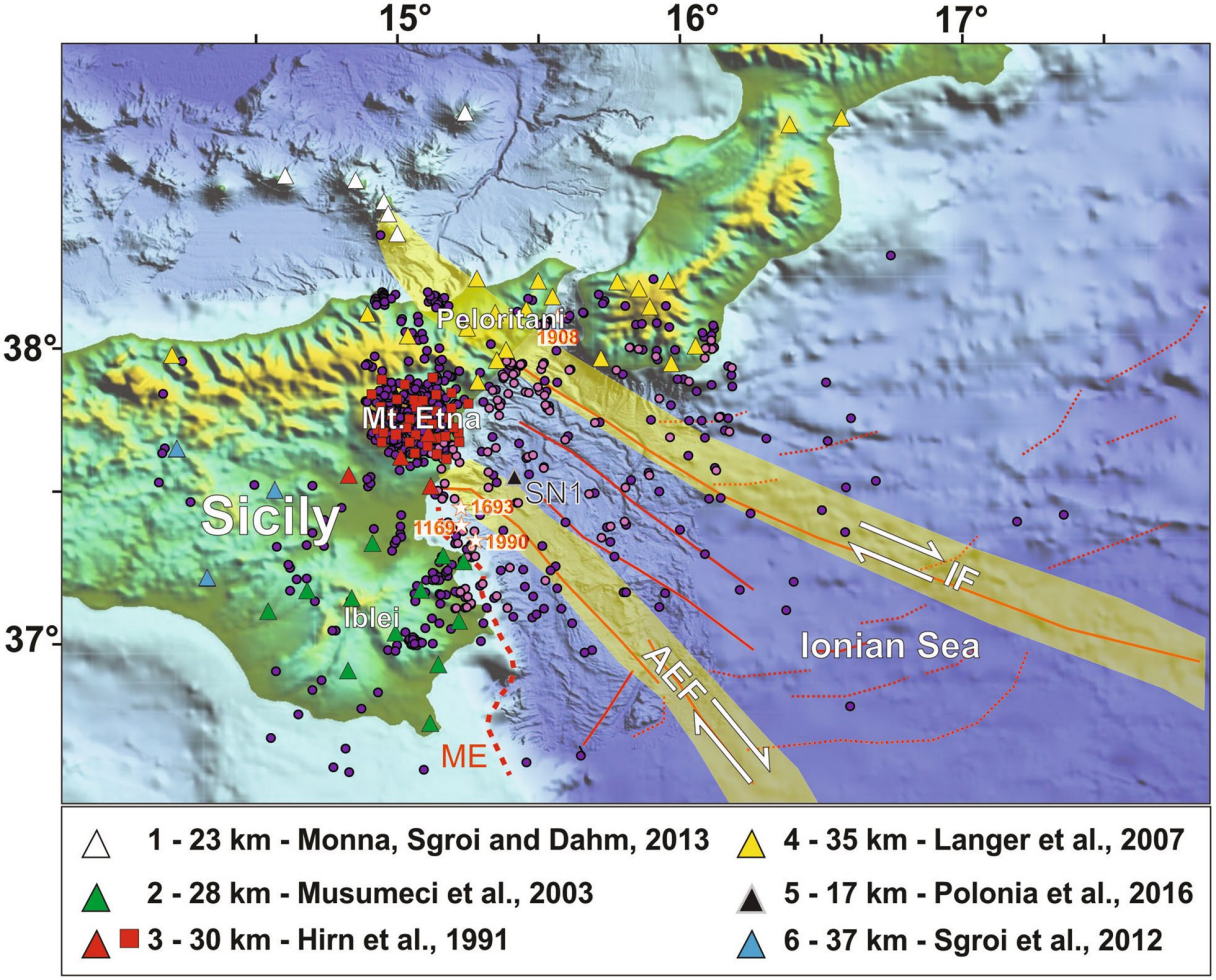
(**a**) Shaded-relief morphological map of the Western Ionian Sea and surrounding with indicated: main geological features, including Alfeo–Etna Fault (AEF) and Ionian Fault (IF) systems^11^, and Malta escarpment (ME); seismic stations used in this work (filled triangles: RSN; red squares: ERN). Colours of triangles and squares are related to the associated velocity models for the offshore-onshore south Calabria and Sicily (see legend for authors); violet and lilac dots indicate the preliminary relocations of 1020 earthquakes occurring in the study area during the deployment of the NEMO-SN1 seafloor station. Violet dots indicate the 912 (on 1020) earthquakes relocated through integration of handpicked travel times recorded by land stations and by NEMO-SN1; lilac dots are the selected earthquakes (108 on 1020) used to compute the new 1D velocity model for the Western Ionian Sea. The epicentres of the four highest magnitude earthquakes ever recorded in Italy^21^ (1169, Mw 6.6; 1693, Mw 7.4; 1908, Mw 7.2; 1990, Mw 5.7) are indicated with white stars and years. Morphobathymetric data are from Global Bathymetry and Elevation Digital Elevation Model: SRTM30_PLUS v8 (https://data.gov.au/data/dataset/global-hillshading-from-srtm30_plus-v8-0-nerp-te-13-1-eatlas-source-ucsd/). The map was compiled using GMT package (version 6.0.0; https://www.generic-mapping-tools.org/), and the image was edited using Adobe Illustrator (CS6; https://www.adobe.com/).

The western Ionian Sea and the Eastern Sicily margin (Fig. 1) are actively deforming area^1–5^, which encompass different geodynamic domains, including Mesozoic rifted margins and the Calabrian Arc subduction system along a geometrically complex Africa/Eurasia plate boundary. Shortening and uplift in the accretionary wedge is driven by plate convergence and retreat of the Tethyan slab^3,6–8^. Rollback processes produce the southeastward migration of the Ionian slab, magmatism, and plate boundary segmentation probably occurring along weak zones inherited from the Tethys ocean^9^.

The Ionian Sea is bounded to the west by the Malta Escarpment (ME; Fig. 1), a regional NNW-SSE-trending, mostly submerged morpho-tectonic domain, affected by recent faulting north of Siracusa (e.g.,^1,10^). In the deep basin, two major oppositely dipping fault systems (Fig. 1), the NW–SE-striking transtensional Ionian Fault (IF) and the Alfeo-Etna Fault (AEF), have been described based on analysis of multiscale geophysical data^3,11^. Since these faults systems are regional deep-seated structures, they are likely candidates as seismogenic sources for the large magnitude earthquakes occurred in the western Ionian Sea during historical times^4,12,13^ as also suggested by the analyses of the seismically triggered turbidite records^14,15^. However, the geodynamic significance and relationships of these structures with subduction processes are still enigmatic, and subject of much current debate. Particularly critical to this point is the lacking of in situ seismological observations in the Ionian offshore, which hampers a more reliable definition of their kinematics and horizontal and vertical continuity, and the compilation of adequately constrained velocity models, which limits the accuracy of earthquake location procedures.

The onshore and offshore portions of Eastern Sicily margin are very heterogeneous, and characterized by different crustal and seismotectonic domains. To date, based on observations carried out using onshore stations and wide-angle seismic data, several velocity models have been proposed^11,16–20^. These models, summarized in Fig. 1, are characterized by different Moho depths, spanning from 17^11^ to 37 km^19^, according to the analyses of different datasets.

The eastern Sicily/Calabria margins have been struck repeatedly by high magnitude earthquakes during historical times, including the largest events ever recorded in Italy^21^ (e.g., 1169, Mw 6.6; 1693, Mw 7.4; 1908, Mw 7.2; 1990, Mw 5.7; Fig. 1), but location and geometry of tectonic sources are still uncertain, also for recent events such as the 1908 Messina earthquake^22^.

Available seismological observations show that the submarine sector of the Calabrian Arc is characterized by small magnitude, scattered earthquake patterns. This should be due to slow deformation rates, but also to the lacking of data due to the poor coverage of the seismic network towards the offshore, where most active deformations are expected. The deployment of an Ocean Bottom Seismometers/Hydrophones (OBS/H) network could thus represent the best strategy to improve observation and monitoring of seismogenic faults in the Ionian Sea.

To date, several seismological experiments have been carried out using temporary network^23–25^, starting with the deployment of the NEMO-SN1 seafloor observatory in 2002, about 25 km offshore of the Eastern Sicily coast in 2100 m water depth (Fig. 1) as a node of EMSO, the European research infrastructure for seafloor and water column monitoring (www.emso.eu)^26^.

In this work, we used data collected by the NEMO-SN1 seafloor observatory between October 2002–February 2003 and June 2012–May 2013, in conjunction with data from the onshore seismic networks, to gather more accurate information on seismogenic structures in this key area of the Mediterranean Sea. Earthquakes recorded by NEMO-SN1 were analysed through different steps, which included: (1) preliminary location of 1020 events recorded by NEMO-SN1 and reconstruction of a new 1D velocity model; (2) relocations of about 2700 events using the new 1D model and a regional 3D model available in the literature; (3) computation of fault plane solutions, to identify the kinematics of observed tectonic structures; (4) tomographic inversion (starting from the new 1D model) to highlight the offshore velocity structures.

## Data analysis

### The new 1D crustal velocity model

**Figure 2:**
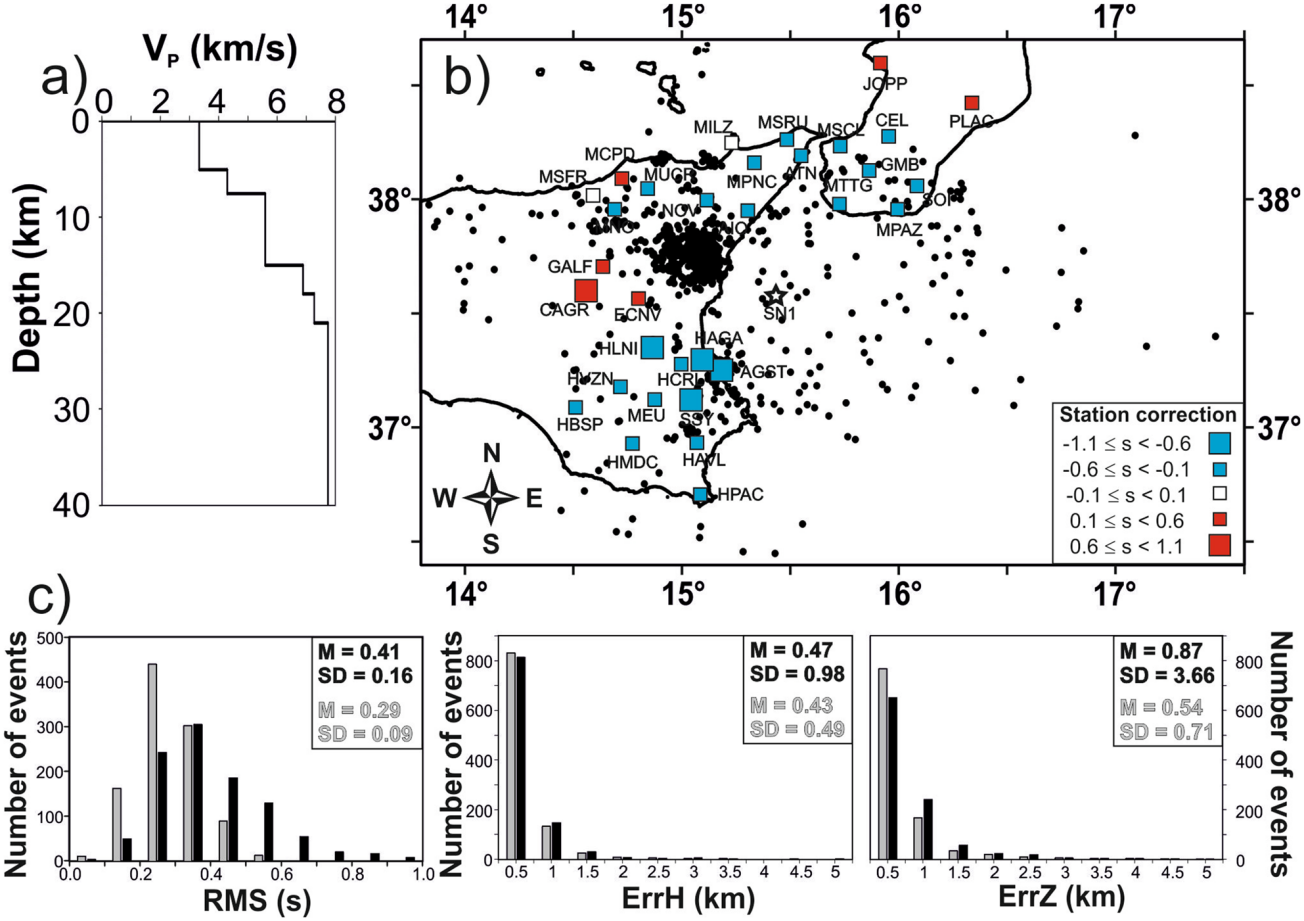
(**a**) The new 1D velocity model (black line) computed with seismological data recorded by NEMO-SN1 seafloor observatory. (**b**) Final relocations of earthquakes using the new 1D velocity model improved by NEMO-SN1 data. Squares indicate the station corrections computed for the 33 seismic stations used in the computation of the new velocity model: low velocities (red squares) are found in Central Sicily and Mt. Etna, associated with soft-sediment covers and with the presence of important thermal effects due to volcanism; high velocities (blue squares) are found in the Hyblean foreland and in the northern Sicily, due to the presence of carbonate and shallow crystalline rocks respectively. (**c**) Quality statistics and comparison between preliminary (black) and final (grey) relocations in terms of Mean (M) and Standard Deviation (SD) values computed on RMS, horizontal (ErrH), and vertical (ErrZ) errors. The presence of an offshore station, although single, contributed to an improved relocation quality. The map was created using the software Surfer (Version 8.09.2391; http://www.goldensoftware.com/products/surfer). The plot was edited using Corel Draw 2018 (Version 20.0.0.633; http://www.corel.com).

A new 1D velocity model for the Western Ionian Sea was compiled using a dataset constituted by 108 out of 1020 earthquakes recorded by NEMO-SN1 and land stations (Fig. 1). The model consists of six layers with the Moho located at 21 km (Fig. 2a). Thickness and velocity of crustal layers are consistent with structural interpretation based on analysis of seismic reflection profiles in the study area, which show a Moho at about 18–20 km^11^. The epicentres of 1020 relocated earthquakes are shown in Fig. 2b, along with related location parameters listed in Table S1. The comparison between preliminary (Fig. 1) and final (Fig. 2b) relocations shows significant differences in terms of RMS, as well as horizontal and vertical errors. The presence of a single seafloor station (NEMO-SN1) and the adoption of a properly constrained velocity model in the earthquake relocation procedure, allowed us to significantly decrease the Mean (M) and Standard Deviation (SD) values computed on RMS, horizontal (ErrH) and vertical (ErrZ) errors (Fig. 2c).

### Earthquake distribution after 1D and 3D relocations

**Figure 3:**
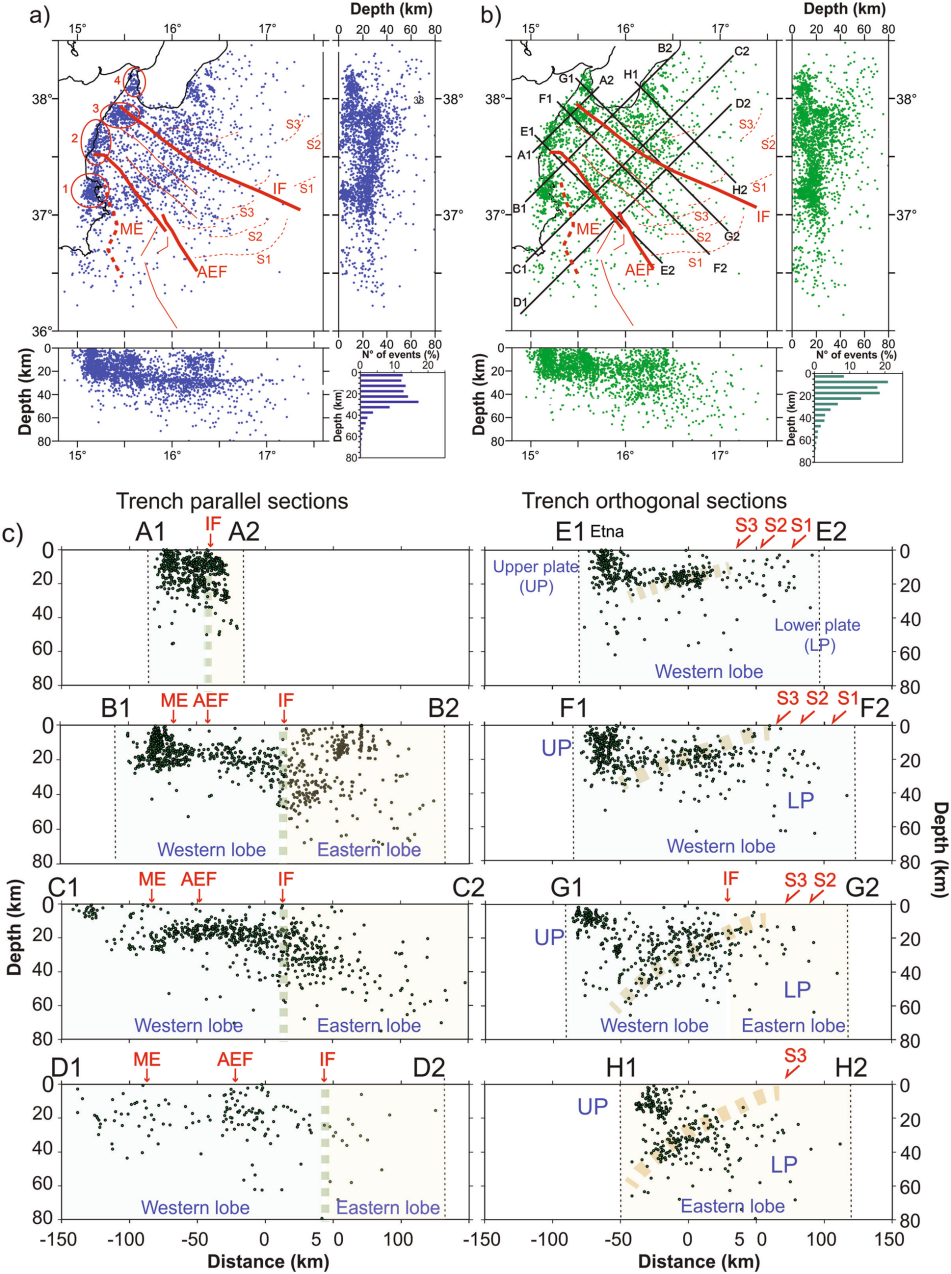
(**a**) Map of relocated seismicity (relocations based on the new 1D velocity model associated to NEMO-SN1) of 2658 earthquakes recorded in the period 1990–2018. This new dataset includes 138 events located with travel times recorded by the NEMO-SN1 station. Earthquake clusters (red circles with letter) are described in the text. Main geological features including Alfeo–Etna Fault (AEF) and Ionian Fault (IF) systems^11^ and Malta escarpment (ME) are sketched in red. Histograms show the distribution of earthquakes up to 80 km depth. E–W and N–S sections show that the shallowest events (down to 20 km) are concentrated on land and in the coastal area. Deeper events (down to 50 km) prevail in the Ionian basin. (**b**) Earthquake relocations based on a previous 3D velocity model of the same dataset shown in (**a**). (**c**) Cross-sectional view of the relocations from the 3D model. Seismicity is projected along the traces shown in (b), having width of ± 10 km from the cross-section line. Red arrows indicate the position of Malta escarpment (ME), Alfeo-Etna fault (AEF), Ionian fault (IF) and splay faults (S1, S2, S3). Maps and sections were created using the software Surfer (Version 8.09.2391; http://www.goldensoftware.com/products/surfer) and edited using Corel Draw 2018 (Version 20.0.0.633; http://www.corel.com).

The compilation of a new 1D velocity model allowed us to reconstruct crustal thickness in the study area. The Moho depth derived from this analyses was used to interpret the observed seismicity in terms of lower and upper plate interaction processes. A further step was to insert results of our analysis in a regional perspective, considering seismicity located both offshore and in the coastal areas, increasing the number of analysed seismic events and extracting the travel times of earthquakes from the ISB catalogue^27^, integrated with the catalogue of INGV—Catania, in the period 1990–2018^28^. We focused on 2658 earthquakes with depth < 80 km, of which 138 earthquakes recorded from NEMO-SN1. A map of earthquakes relocated in the study area is shown in Fig. 3, which includes depth distribution (right bottom histogram) and sketched E-W and N-S sections (hypocentre parameters of 1D relocations are reported in Table S2) for earthquake relocations using the 1D (Fig. 3a) velocity model.

The 1D relocation shows a non-homogeneous distribution of events with a tendency of forming discrete clusters. We observe a major concentration of earthquakes around the Mt. Etna, where seismicity is associated to active volcanism, such as the vigorous Etna eruption occurred from October 27, 2002 to January 30, 2003, accompanied by several hundreds of events^29,30^, most of them also recorded by NEMO-SN1^31^. We note that seismicity in the Ionian basin is rather diffused, with earthquake concentration in four main clusters (Cluster 1 to 4 in Fig. 3a) along the eastern Sicily coastline and in key areas such as in the south-eastern Calabria coastline. Cluster 1 is located in the Hyblean region, on the footwall of the Malta Escarpment (ME); Cluster 2 is located along the Etna volcano underwater flank; and Clusters 3 and 4 are localised on the northern and southern blocks of the IF.

Since we were analyzing a large seismic dataset that is spread over areas with different crustal structure, we were forced to relocate seismic events also considering a regional 3D model^32^, which averages local variations in crustal thicknesses. This model is derived from a detailed 3D image of the Calabro-Ionian subduction system, and shows a crustal thickness of about 25 km in correspondence of the western Ionian Sea. The map of seismic events together with E–W and N–S sections and the histogram of depth distributions is sketched in Fig. 3b (hypocenter parameters of 3D relocations are reported in Table S3). Differences between 1D and 3D locations were evaluated in terms of differences in latitude, longitude and depth (Fig. S1). We note that relocations performed using the two velocity models are nearly identical in plan-view (latitude and longitude differences below 8 km for about 94% and 91% of all events) whereas hypocentre variations are larger, reaching even 16 km (Fig. S1). Two sets of vertical cross sections, orthogonal to the transverse fault systems (Fig. 3c sections A–D) and orthogonal to the subduction thrust (Fig. 3c sections E–H) were selected to highlight relationships between earthquake distribution and major structural features. The trench parallel sections of Fig. 3c shows a major boundary in correspondence of the IF. East of the IF (Eastern Lobe of the accretionary wedge according to Polonia et al.^3^), the seismogenic layer is about 70–80 km thick, while to the west of it (Western Lobe), seismicity is shallower, reaching a maximum depth of about 20–30 km. The region between the ME and the IF (inner accretionary wedge) in sections B and C (Fig. 3c) shows a rather flat alignment of earthquakes at about 18–20 km depth, with very limited earthquakes in the uppermost 10 km, suggesting that the accretionary wedge in this region does not host seismic activity. In section D (Fig. 3c), between 0–10 km of depth, the wedge shows very limited seismic activity as well, with the exception of the area where the AEF intercepts the Alfeo seamount, constituted by Mesozoic carbonate rocks^33^. Thus, the AEF shows an aseismic behaviour in the weak accretionary wedge (no seismicity associated with the AEF in sections B and D), and earthquake activity around the Alfeo seamount, suggesting that changes in rheology within the subduction complex represents a primary control on seismic fault behaviour.

The cross sections orthogonal to the subduction direction (sections E–H, Fig. 3c) highlight variations in seismic activity from the upper to the lower plate. In the upper plate (northern segments of the cross sections), seismicity is mainly concentrated in the first 20 km, while moving seaward, seismicity is very low in the upper 10–20 km, with earthquakes concentration along a plane gently dipping towards NW. This alignment may correspond to the boundary between the underplating Africa plate and overriding Eurasia plate. It is worthwhile noting that this NW dipping surface becomes steeper moving from Sicily to Calabria.

Previous investigations based on multichannel seismic profiles and gravity data^3^ documented that the subduction system in the Eastern and Western lobes of the accretionary complex show completely different structural styles and geometries. The Western lobe is similar to a typical accretionary wedge detaching on the base of the Messinian evaporites in the frontal part (southeast of splay faults S1, S2, S3 in Fig. 3c) and on the (crystalline?) basement in the inner region, northwest of a series of splay faults (S1, S2, S3 in Fig. 3c). Conversely, the Eastern Lobe shows higher uplift and deformation rates, and resembles more closely a thrust and fold belt where the crystalline basement is involved in deformation (thick skinned tectonics^34^). This new seismological evidence confirms this interpretation, showing that seismicity in the Eastern Lobe is deeper than in its western counterpart, suggesting a link between deep processes and shallow structural development.

### Focal mechanisms

The handpicking of the seismic phases allowed us to collect P-wave first motion polarities and compute focal mechanism solutions. We considered polarities of all events occurred in the time period between 1990 and 2018. Out of 223 new focal mechanisms (1.6 ≤ M ≤ 4.7; Table S4), 66 were computed using polarities detected from the NEMO-SN1. We define the quality factor (Q) of focal solutions, depending on the degree of polarity misfit and on the range of uncertainties of strike, dip, and rake values. Q ranges from 0 (low quality) to 2 (best quality). In our dataset, 69, 139, and 15 solutions have Q = 2, 1, and 0, respectively.

**Figure 4:**
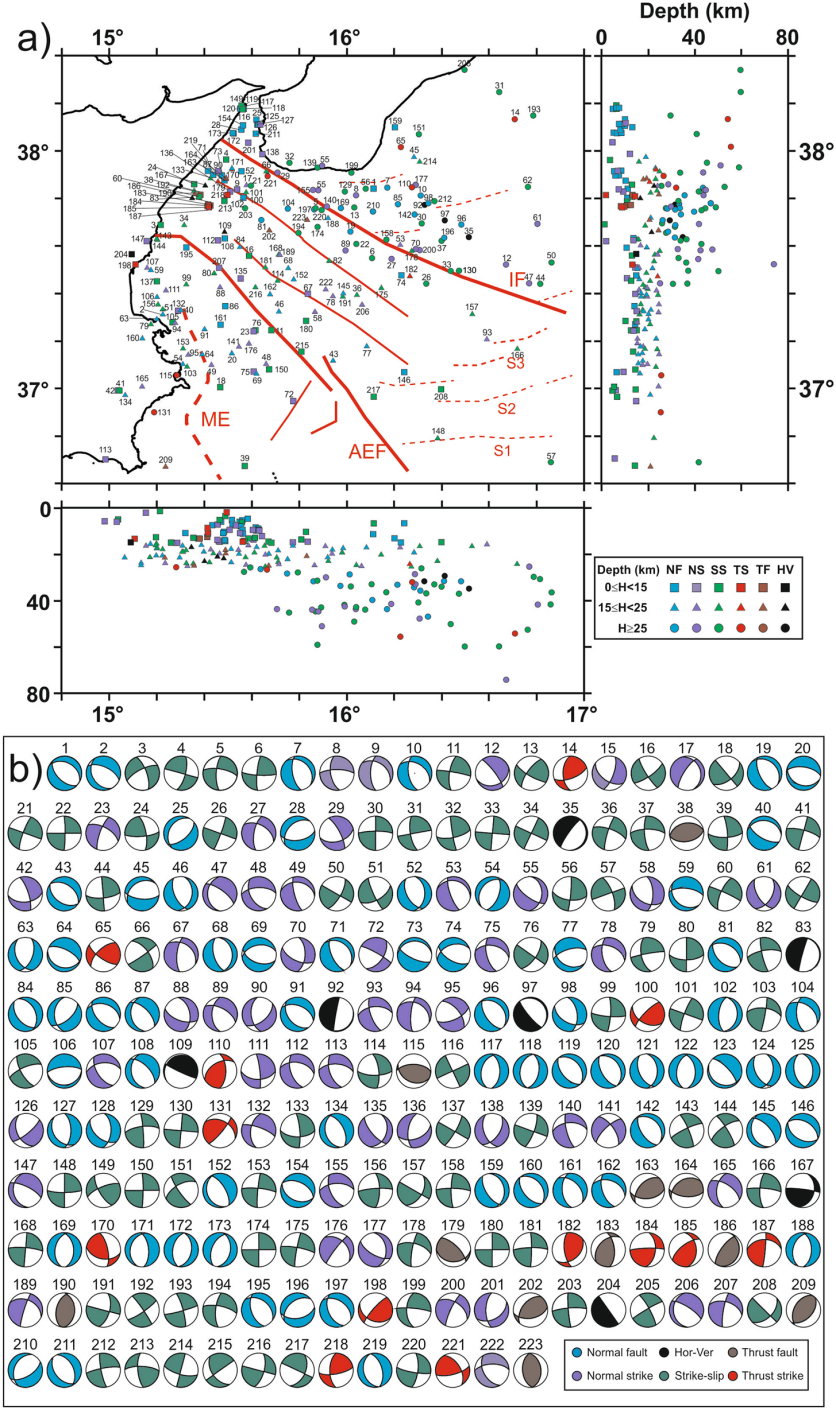
(**a**) Location map and EW and NS sections for the new 223 focal mechanisms computed in this study. The different colours of the epicenters refer to the kinematic classification^35^ based on the plunge of T and P axes (Fig. S2). Squares, triangles and circles indicate the three depth ranges of events (0–15 km; 15–25 km; > 25 km; see legend in the bottom right inset). Main geological features including Alfeo–Etna Fault (AEF) and Ionian Fault (IF) systems^11^, Malta escarpment (ME) and splay faults (S1, S2, S3) are sketched in red. (**b**) The new 223 focal solutions computed in this work. Map and sections were created using the software Surfer (Version 8.09.2391; http://www.goldensoftware.com/products/surfer); (**a**,**b**) were edited using Corel Draw 2018 (Version 20.0.0.633; http://www.corel.com).

Following a classical classification scheme^35^, based on the plunge of T-, B-, and P-axes, we subdivided our solutions into five kinematic categories: thrust, thrust-strike, strike, normal, normal-strike, and a type of mechanism having horizontal (or vertical) axes. These categories are schematically represented in a ternary diagram (Fig. S2) with different coded colours. Despite the presence of a few events having thrust and thrust-strike kinematics, most of the considered earthquakes have a prevalent normal, normal/oblique, and strike-slip kinematics. The relocated epicentres are projected onto the map of Fig. 4a, with symbols (squares, triangles and circles) referring to three depth ranges (0–15 km, 15–25 km, and > 25 km) and colours referring to the prevalent kinematics of the classification scheme^35^ (Fig. 4b).

**Figure 5:**
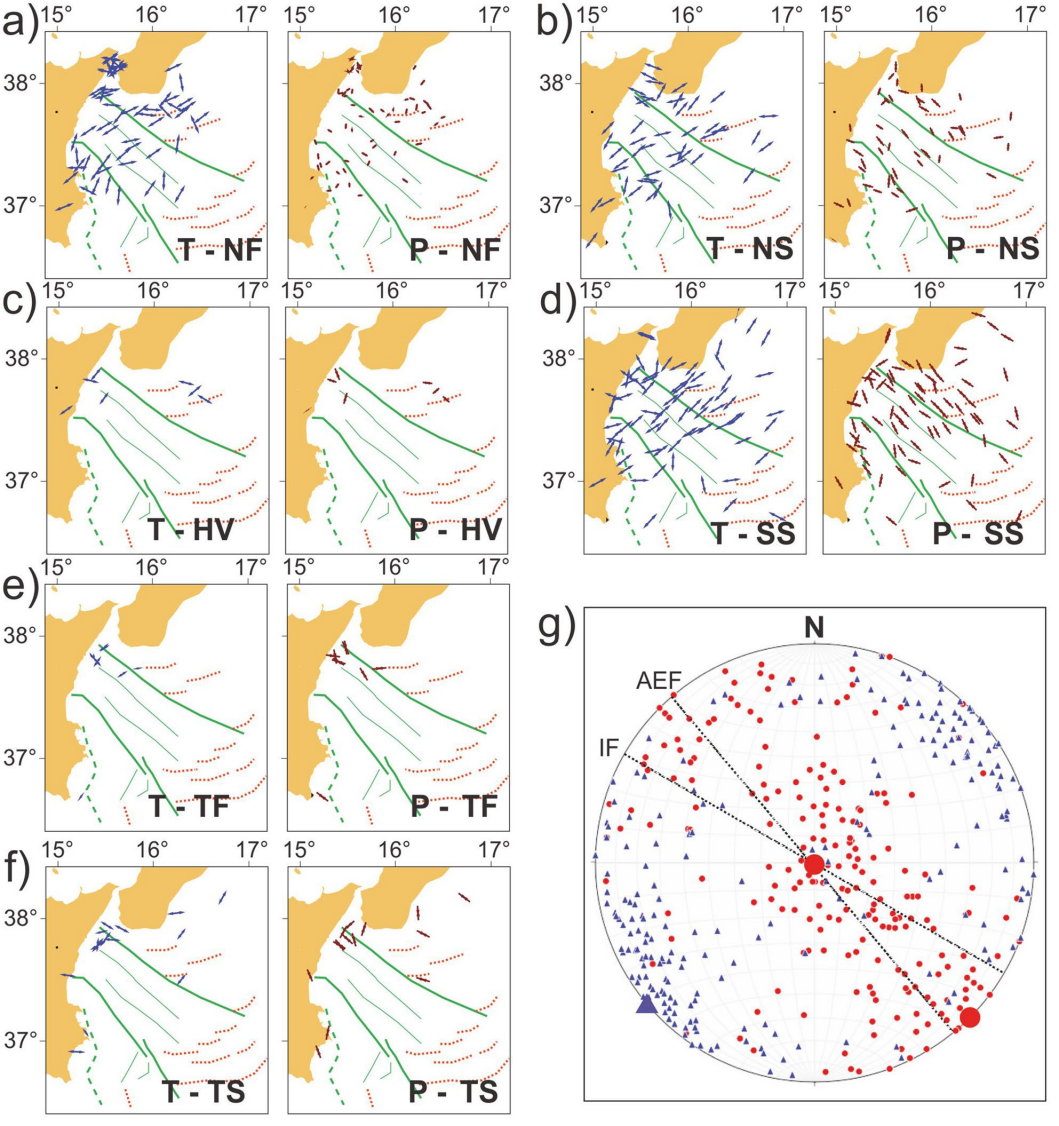
Maps of the orientation of P- and T-axes subdivided in normal fault (**a**), normal-strike (**b**), horizontal-vertical (**c**), strike-slip (**d**), thrust fault (**e**), and thrust-strike (**f**). Synoptic polar diagram of P- and T-axes (**g**). Maps are compiled using GMT package (version 6.0.0; https://www.generic-mapping-tools.org/) and image was edited using Adobe Illustrator (CS6; https://www.adobe.com/).

Maps of focal mechanisms and related horizontal components of P- and T-axes (Fig. 5a–f) as well as the synoptic polar diagram of P- and T-axes (Fig. 5g) show that two active tectonic regimes presently coexist in the Ionian Sea: -an arc-parallel extensional regime (with a sub-vertical P-axis and a NE–SW-trending T-axis); -a strike-slip regime (with a sub-horizontal NW–SE-trending P-axis and a sub-horizontal NE–SW-trending T-axis) across the Calabrian accretionary wedge. This observation is highlighted by density contours of P- and T-axes in Fig. S3. Maps of Fig. 5 show that both regimes are active over most part of the accretionary wedge, particularly along the main fault zones (i.e. Alfeo-Etna and Ionian faults). The angular relationships between these two faults and the P- and T-axes of earthquakes show that both systems are undergoing a regional transtensional regime, as also reported by recent seismological analyses (e.g.,^36^).

### Tomography

**Figure 6:**
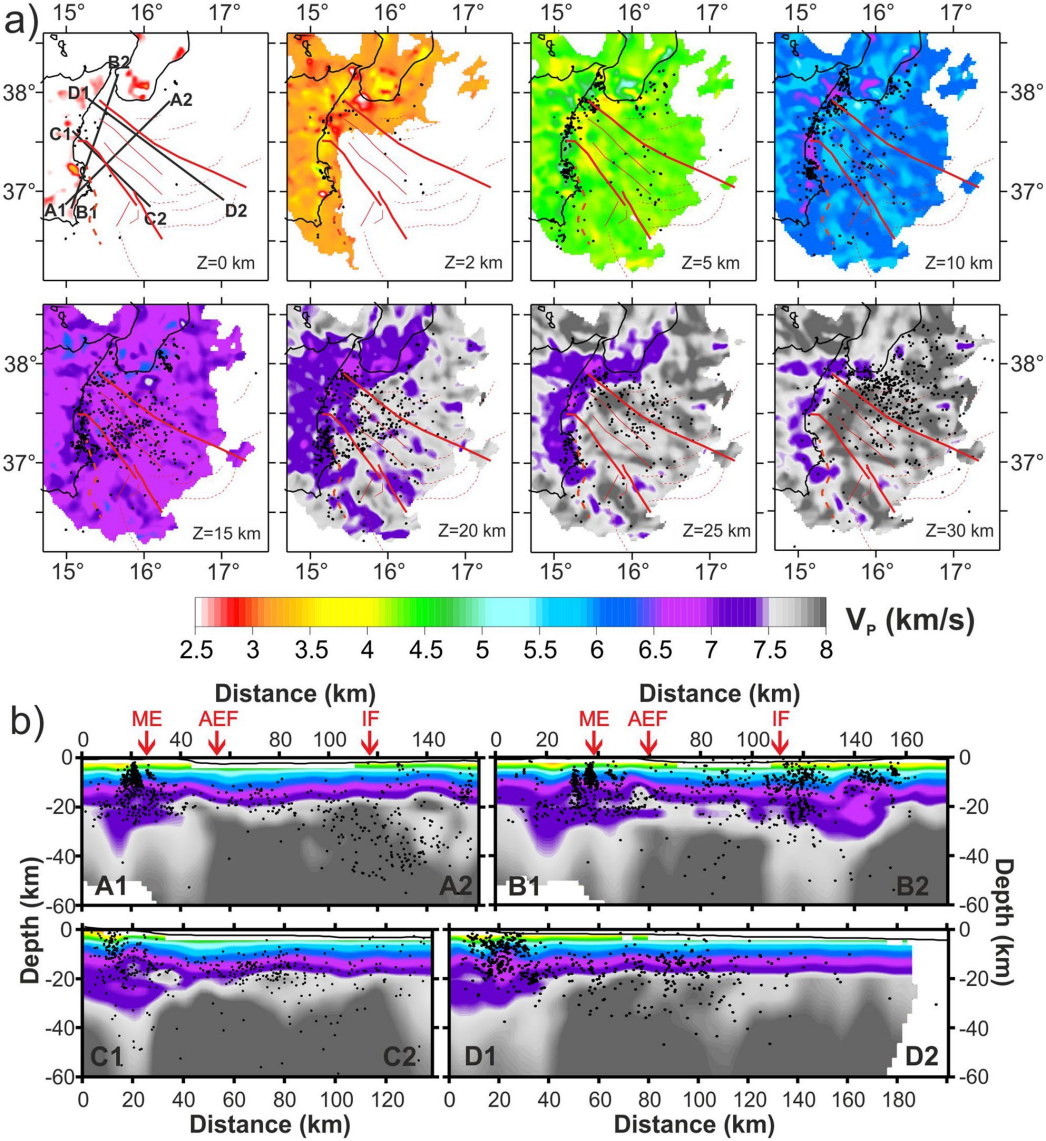
(**a**) Tomography layers of V_P_ velocity model obtained by real data inversion, using, as starting model, the new 1D velocity model computed for the Ionian Sea. The area that is not sampled is white. Black lines in the 0 km layer are for profile tracks. Black dots are earthquakes relocated using the tomoDDPS and the 3D velocity model^32^. (**b**) Profiles of V_P_ velocity model. Profile swath width is within ± 10 km from the profile track. Red arrows on profiles A1-A2 and B1-B2 indicate the position of Malta escarpment (ME), Alfeo-Etna fault (AEF) and Ionian fault (IF). Maps and sections were created using the software Surfer (Version 8.09.2391; http://www.goldensoftware.com/products/surfer) and edited using Corel Draw 2018 (Version 20.0.0.633; http://www.corel.com).

A tomographic inversion was performed to explore the crustal structures of the western Ionian Sea. We started from the whole set of recorded submarine earthquakes (2742 events), which allowed us to investigate the tectonic structure with unprecedented detail compared to previous studies (e.g.,^32,37^). Figure 6a shows the maps of tomographic layers, whereas Fig. 6b includes tomographic sections having trends nearly perpendicular and parallel to the AEF and IF directions. All results, including the tomographic map, the E–W and N–S cross-sections of P wave ray tracing (Fig. S4) and the checkerboard synthetic tests (Fig. S5) computed on the same layers and cross-sections, are shown in Fig. 6. They confirm that the crustal structure of the study area is well resolved down to about 40 km depth, whereas below 40 km and in the outermost sectors, the resolution and accuracy are limited by the event-station geometry and the poor coverage of seismic stations.

The tomographic images (Fig. 6a) show the presence of an arc-shaped area of low V_P_ connecting the ME and IF near the eastern coast of Sicily at different depths. Discontinuous patches, characterized by lower seismic velocities between 10 and 20 km of depth, correspond to the main transverse structures (i.e., the AEF and the IF) segmenting the subduction complex^38^. Shape and location of this area are consistent with the distribution of the depth differences shown in Fig. S1. Relevant features of the model are also evident in cross-sections (Fig. 6b), with a thicker crust (up to 30 km) associated to the seismic clusters near the coast and along the IF system. In the central part of the study area, crustal thickness is about 20 km on average, a value compatible with the presence of an oceanic crust^39^.

## Discussion

The seismogenic behaviour of the subduction faults in the Calabrian Arc is largely unknown, due to the lacking of thrust-type earthquakes during historical times in the forearc region. Our new dataset confirms the paucity of compressional earthquakes, except for those observed at the northern tip of the Ionian Fault (Figs. 5 and 6), and for some events with horizontal axis offshore southern Calabria, in the vicinity of out-of-sequence thrust faults located in the inner accretionary wedge (Fig. 4a).

The observed small number of subduction-type earthquakes could be consistent with a few hypotheses: (1) the compressional deformations in the Ionian Sea are active but non- or poorly seismogenic due to the presence of lubricating “soft” evaporites in the outer accretionary wedge^2, 11^ and/or a weak subduction interface; (2) the Calabrian accretionary wedge is substantially inactive, although incipient deformations were observed along the frontal wedge^34^; (3) the frontal (southern) thrusts of the accretionary wedge are locked and presently loading elastic stresses, partly released along strike-slip/transtensional faults and/or along inner (northern) thrusts; in such a case, the frontal thrust may release strong earthquakes in the future; (4) we are observing an incomplete record of earthquakes, since small earthquakes occurring in offshore sectors cannot be recorded by the onshore seismic network.

Such major issues could be addressed only through an integrated seismological, geodetic, and marine geophysical data analysis.

### Subduction processes and transverse faults

Local GPS velocities indicate very low strain rates in the region^40,41^, but there is no clear evidence suggesting that the Calabrian subduction is halted and seismically inactive. For this reason, the presence of locked subduction interface, at least offshore Calabria, where the slab is still attached^7,37^, is not unlikely. In this scenario, finite slip along the subduction interface (both seismic and creeping) might cause sufficient stress changes in the overlying crustal extensional faults to generate large earthquakes in a relatively short time interval. Seismic coupling of the sub-horizontal and weak basal detachment covered with over 10 km of sediments and rocks^3^, may be accommodated along shallower strike-slip and/or normal faults. This seismogenic behaviour was described in other regions worldwide, such as during the Mw 8.8 Maule, Chile^42^ and the 2011, Mw 9.0, Tohoku-Oki, Japan^43^ earthquakes, where seismic or aseismic slip on the subduction interface can trigger extensional earthquakes in the forearc.

Our seismological dataset highlights extensional earthquakes deeper than crustal levels, at least in the IF region, implying that such lithospheric discontinuity is segmenting the basal detachment. Seismic reflection profiles, in fact, show that the Western Lobe of the subduction system is detaching on the base of evaporites (6 km depth), while in the Eastern Lobe the detachment is located on top of the basement, at about 10 km depth^11^, and this change occurs in correspondence of the IF. This along-strike margin segmentation reduces the hazard related to the subduction interface itself, but points for the need of a better hazard assessment related to the transtensional fault systems.

### Shortening and arc orthogonal extension

Arc-parallel extension along curved subduction zones are rather common features on Earth. Examples include the Aleutian arc^44^, the High Himalaya and Tibet^45,46^, Lesser Antilles^47^, and the Ryukyu arc^48^. In Italy, the same features are observed in northeastern Calabria^49^. Models for such deformation imply strain partitioning in complex convergent zones due to, for instance, lateral extrusion, oroclinal bending, radial spreading, or oblique convergence, but also to locking of the main frontal/basal thrust and a consequence of partitioning within the accretionary wedge^45^. In the western Ionian Sea case, we do not have any compelling evidence pointing towards one of the aforementioned models to account the observed arc-orthogonal extension. Conversely, our new data, analysed considering the presence of a major volcano (Mount Etna) and the evidence for serpentinite diapirism within the external subduction system^9^, suggest that arc-orthogonal extension might occur along lithospheric faults reaching down to the lower plate mantle. Analyses of new focal mechanisms and distribution of P- and T-axes provides clues for such interpretation. Maps of focal mechanisms and related horizontal components of P- and T-axes (Fig. 5a–f), as well as the related synoptic polar diagram of P- and T-axes (Fig. 5g) show that two main active tectonic regimes presently coexist in the western Ionian Sea: a strike-slip domain, with a NW–SE-trending P-axis and a NE–SW-trending T-axis, across the Calabrian accretionary wedge; and a wedge-parallel extensional domain, with a sub-vertical P-axis and a NE–SW-trending T-axis. Both regimes are active over most part of the accretionary wedge, and seismic distribution correlates well with the main transverse fault system, whose orientation and geometry are in good agreement with this reconstruction (Fig. 5g). It is worthwhile noting that coexisting orogen-parallel strike-slip and orogen-normal extensional tectonic regimes are observed in areas where frontal thrusts become temporally locked and contractions can therefore be accommodated through strike-slip and normal faults (e.g.,^50^).

GPS data and previous observations on seismicity suggest that the subducting African plate may contain several active fault/shear zones separating Apulia from Adria and the Hyblean plateau^41^. In this framework, a key role could be played by the submerged Calabrian Arc subduction system in the Ionian Sea, described either as part of the Hyblean-Malta block or as part of the diverging Apulian block moving towards the northeast, relative to Europe^51^. Our seismological observations indicate that the major transverse fault systems segmenting the accretionary wedge, AEF and IF, are crustal/lithospheric boundaries accommodating transtensional deformation, in agreement with the geodetic divergence of the Hyblean and Apulian blocks^52^, which should cause a deep fragmentation of the subduction system. This is also confirmed by the different seismogenic behaviour of the Eastern and Western lobes revealed by our new dataset (Fig. 3b,c). Moreover, crustal velocity models account for a Moho depth decreasing from about 35 km in the Calabrian domain, to 15/20 km in the western Ionian Sea^8,53^. A shoaling of the Moho depth between the AEF and IF was also observed in seismic reflection profiles, showing the collapse of the accretionary wedge along NW–SE lithospheric faults^9^. All these evidences suggest asymmetric rifting affecting a large corridor between the two transverse fault systems, that may have occurred (and still occurs) as a response to the Calabrian slab dynamics and/or in response to plate divergence driven by tectonic rotations. If the corridor between AEF and IF represents an area of incipient rifting, elastic properties of the lithosphere should be marked by P- and S-wave velocity anomalies. The map displayed in Fig. S1 should be considered a further evidence of such an occurrence (thinner lithosphere and rifting processes), since it highlights regions where the Moho is shallower than 25 km. In addition, tomographic maps highlight an arc-shaped velocity anomaly connecting AEF and IF (Fig. 6a).

### The source region of the 1908 Messina earthquake

**Figure 7:**
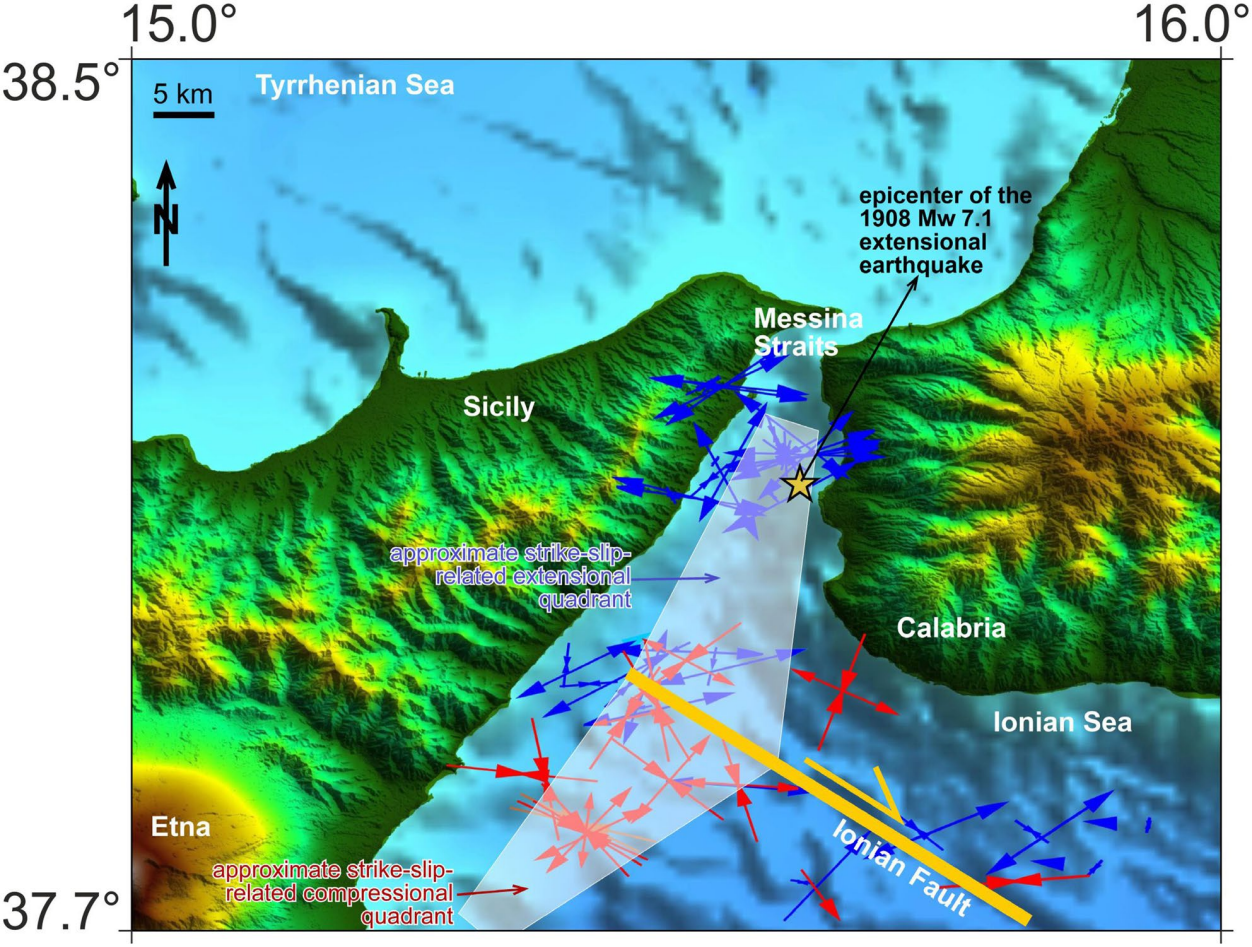
Map of the Messina Straits area with the northwestern termination of the right-lateral strike- to oblique-slip Ionian Fault. In this area, most extensional earthquakes (see the related T-axes in blue) are located in the northern extensional quadrant of the fault tip, whereas most compressional earthquakes (see the related P-axes in red) are located in the southern compressional quadrant of the fault tip (e.g.,^54^). Hence, we propose that the ongoing extensional regime in the Messina Straits and related earthquakes (including the 1908 one) may at least in part be driven by the right-lateral displacement along the Ionian Fault rather than by the regional stress field. The epicenter of the extensional 1908 earthquake is from Michelini et al.^69^. Morphobathymetric data are from Global Bathymetry and Elevation Digital Elevation Model: SRTM30_PLUS v8 (https://data.gov.au/data/dataset/global-hillshading-from-srtm30_plus-v8-0-nerp-te-13-1-eatlas-source-ucsd/). The plot was edited using Corel Draw 2018 (Version 20.0.0.633; http://www.corel.com).

Analysis of the focal mechanisms indicates the presence of two individual patches in the Messina Straits and offshore Taormina, about 40 km to the south-southwest along the coast, respectively, at the opposite sides of the north-western tip of the NW–SE-striking right-lateral Ionian Fault (Fig. 7). This spatial distribution might indicate that the extensional tectonics within the Messina Straits (most probably including the 1908 Mw = 7.1 earthquake) may be triggered by the right-lateral displacement along the Ionian Fault (see “wing” or “horsetail tip” structures^54^; Fig. 7). This hypothesis, to be confirmed by other independent evidence, would indicate a main role played by deformation along the IF in generating large magnitude earthquakes in the Messina Straits area, prone to high seismic hazards.

## Conclusions

The Calabrian Arc (CA) accretionary prism in the wester Ionian Sea is crossed by NW–SE striking deeply rooted faults, whose geodynamic significance is still enigmatic since seismological observations are scant. This implies the lacking of adequately constrained velocity models and large errors in the earthquake location procedures. Recent observations carried out with a submarine observatory (NEMO-SN1) were used to overcome these limits, and obtain more reliable seismological data for this region. Compilation of earthquake distribution maps and tomographic models led us to draw the following conclusions.

- Spatial changes in the depth distribution of earthquakes highlight that the Ionian Fault (IF) is a major tectonic boundary along which thickness of the seismogenic layer varies from 20–30 km to the West, to more than 70 km offshore southern Calabria.
- The Alfeo-Etna Fault (AEF) shows an aseismic behaviour in the accretionary wedge and a cluster of earthquakes close to the Alfeo seamount, suggesting that changes in rheology within the subduction complex represent a primary control on seismic fault behaviour.
- The lithosphere W of the Ionian Fault is affected by transtensional stresses. Maps of focal mechanisms and related horizontal components of P- and T-axes, show that a NW–SE strike-slip regime coexists with wedge-parallel extensional deformations.
- Seismic activity varies orthogonally to the subduction direction. In the upper plate, seismicity is mainly concentrated in the upper 20 km while moving trenchward it is concentrated along a NW gently dipping plane. This pattern may correspond to the boundary between the underplating Africa and overriding Eurasia plates, and it appears steeper offshore Calabria.
- A small number of subduction-type earthquakes suggest two main hypotheses: shortening is active but non- or poorly seismic; the frontal thrusts of the accretionary wedge are locked and actually loading elastic stress, possibly partly released along strike-slip/extensional faults and inner thrusts.
- The observed spatial distribution of earthquakes in the Messina Straits region indicates that extensional tectonics may be caused by right-lateral displacement along the Ionian Fault, providing new insights on seismogenesis of the 1908 Messina earthquake.

## Data and methods

### Data

The NEMO-SN1 seafloor observatory (Multidisciplinary Oceanic Information SysTem MOIST—http://moist.rm.ingv.it/) was deployed in the Ionian Sea, offshore Catania (Fig. 1a). It was fully working at 37.442 N, 15.393 E, and 2072 m water depth during October 2002–February 2003 (operating in autonomous acoustic-linked mode^23,31^), and at 37.548 N, 15.398 E and 2037 m of water depth during June 2012–May 2013 (operating in cabled mode^55,56^) (http://www.moist.it/sites/western_ionian_sea/2). NEMO-SN1 was equipped with a set of geophysical and oceanographic instruments including: gravitymeter, hydrophone, Conductivity and Temperature versus Depth—CTD, 3-C single-point current meter, several status sensors, and a three component broadband seismometer (Guralp CMG-1 T seismometer, with a 0.0027 to 50 Hz bandwidth frequency response and 100 Hz sampling rate).

Data collected by NEMO-SN1 were integrated with recordings from available seismological catalogues, including local earthquakes recorded by the National Seismic Network (RSN)^57^ and the Etna Regional Network (ERN), both managed by the Istituto Nazionale di Geofisica e Vulcanologia (INGV—INGV Seismological Data Centre and Osservatorio Etneo). In 2002–2003, the RSN was composed by about 25 stations in the study area (S-13 Teledyne Geotech sensor, sampling rate of 50 Hz). In the same years, the Etna Regional Network (ERN), mainly employed for monitoring the Mount Etna seismo-volcanic activity, consisted of about 20 stations distributed around the volcano edifice (3-component stations Mark L4C with a 160 Hz sampling rate). At present, the RSN and the ERN networks have approximately 150 stations deployed in Sicily, Aeolian Islands, and southern Calabria (Fig. 1), all equipped with three-component extended band (Lennartz 5 s) and/or broad-band (Trillium 40 s) sensors^58,59^.

Prior to data analysis, we gathered information on earthquake locations reported by the CSI catalogue^60^, the ISB catalogue^27^ and the *“Catalogo dei Terremoti della Sicilia Orientale – Calabria Meridionale, INGV, Catania”*^28^, collecting a seismological dataset consisting of 1020 crustal and sub-crustal earthquakes during the time-period covered by the NEMO-SN1 records. We handpicked arrival times of the events recorded by the land stations and the NEMO-SN1 seismometer. To standardize the compiled dataset in terms of location procedure and result quality, we performed a preliminary relocation integrating arrival times both from land seismic stations (from both RSN and ERN) and from the NEMO-SN1 seafloor observatory.

### Event location

Earthquake locations were computed using the Hypoellipse code^61^, which allows for application of different 1D velocity models^19^ and takes into account the negative quote of the seafloor seismic station^31^. To standardize the compiled dataset in terms of location procedure and result quality, we performed a preliminary relocation integrating arrival times both from land seismic stations (from both RSN and ERN networks) and from the NEMO-SN1 seafloor observatory by using the Hypoellipse code. Six 1D velocity models were selected from the literature, as representative of the structural heterogeneities in the study area: Model 1 is a crustal model for the southern Tyrrhenian Sea derived from tomographic studies performed with datasets of crustal and sub-crustal earthquakes recorded by a network of land and marine stations^20^ (Moho depth 23 km); Model 2 derives from seismotectonic studies performed in the Hyblean area^17^ and involves a Moho depth of 28 km; Model 3 was compiled for the Etna area^16^ (Moho depth of 30 km); Model 4 was obtained from a simultaneous inversion of hypocenter coordinates and a 1D velocity structure using earthquakes recorded in the NE Sicily and SW Calabria^18^ (Moho depth of 35 km); Model 5 is derived from geophysical and geological data collected in the Eastern Sicily offshore^11^, and assumes a Moho depth of 17 km; -Model 6 derives from a seismotectonic study performed in central Sicily^19^ with a Moho depth of 37 km.

The whole dataset was relocated using a regional 3D velocity model^32^ and the tomoDDPS algorithm^62^. This software has the advantage of using a combination of both absolute and differential arrival times, so that for earthquakes with foci lying close to each other, travel times errors due to incorrect velocity models in the volume outside the cluster will essentially be cancelled. Furthermore, we observed that after five interactions of inversion, the P and S residuals were reduced from about 0.30 s to 0.20 s for P-phase and from 0.43 s to 0.26 s for S-phase (33.3% for P and 39.5% for S). At the end, the algorithm can produce a better clustering of earthquakes and a reduced residuals (RMS) of about 56%, an average of 0.07 s is computed.

### The 1D velocity model

We used the standard VELEST software^63^ for the computation of the 1D velocity model. In this approach, hypocentre locations, velocity structure, and station corrections are derived using a simultaneous inversion of P and S waves. S-wave readings were included to better constrain the earthquake location. Since NEMO-SN1 (the reference station) was placed at − 2100 m b.s.l., a correction for depth of event and quote of the seismic stations was applied. Data inversion involved the use of starting velocity models^64^. We selected the models^11,16^ previously described and used to preliminary locate the events. Moreover, three additional velocity models^53,65,66^ were considered to account for structural heterogeneities and peculiar velocities in the offshore area.

We selected 108 best quality hypocentres out of the 1020 relocated events (GAP ≤ 220°; RMS ≤ 0.5 s; P and S phases number ≥ 8; violet and lilac dots in Fig. 1). In addition to the offshore station, we used 33 seismic stations (squares in Fig. 2b) from adjoining onshore areas, ensuring coverage around NEMO-SN1.

The steps leading to the compilation of a final 1D velocity model consisted of several inversions. For each inversion, we analysed the RMS trends versus the number of iterations, and choose, as the best model, the one corresponding to the minimum misfit of traveltime residuals. In this way, we created some models with thin layers near the Moho discontinuity to constrain its depth and tune the damping factors as to avoid both overdamping and an unrealistic solution. Finally, we considered as final velocity model the output showing the most stable solution. Starting from a misfit of travel-time residuals equal to 1.07 s, we achieve a minimal misfit of 0.19 s.

### Focal mechanisms

We applied the FPFIT standard procedure^67^, considering the polarities of all events in the western Ionian Sea area during the 1990–2018 time period. We selected events with a minimum number of eight clear polarities homogenously distributed over the focal sphere (approximately 60% of events have a number of polarities ≥ 11) and with discrepant polarities ≤ 2 (about 68% of earthquakes do not have discrepant polarities).

### Local tomography algorithm

Seismic velocity modelling was carried out through the program LOTOS^68^, by inverting a total of 24,256 P and 15,741 S arrival times recorded at 160 stations. Prior to the inversion, we defined a set of parameters, in particular, a preliminary guess for the 1D seismic velocities. We used the new 1D velocity model computed for the western Ionian Sea (Fig. 2b) for the crust. We then extended this model down to a depth of 100 km, and fixed the average value of Vp/Vs ratio at 1.73. The grid nodes are installed according to the distribution of rays in the 1D model and were chosen based on the presumed size of anomalies, assumed to be greater than the set of grid nodes. The software is not able to work in absence of rays, whereas in areas of a high ray number it can increase the grid density up to the maximum value that was assumed as 5 km for both horizontal and vertical directions. The solution is controlled by smoothing parameters to reduce the difference in the final values of the neighbouring nodes. To decrease the influence of the grid parameterization, the code repeats the inversion using four grids with different basic orientations (0°, 22°, 45° and 67°). The resulting velocity anomalies obtained from all grids are then combined using the same algorithm, which averages the values in the regular grid. The tomographic inversion was obtained by discarding events with less than 8 observations, and arrival times with residuals of more than 1.5 s for P and 2.5 s for S rays.

## Supplementary Information

Supplementary Information 1.

Supplementary Information 2.

Supplementary Information 3.

Supplementary Information 4.

Supplementary Information 5.

## Data Availability

Waveform data of earthquakes recorded by the INGV stations (land stations and OBS hosted in the NEMO-SN1 marine station) are available online from the European Integrated Data Archive (EIDA) at http://eida.rm.ingv.it. P- and S-phase arrival times associated to earthquakes occurred in the Ionian basin and analysed in this study are available online in the database ISIDe (Italian Seismological Instrumental and parametric Data-base; http://cnt.rm.ingv.it/iside). Metadata from the NEMO-SN1 station are available online at the Multidisciplinary Oceanic Information SysTem (MOIST) at http://moist.rm.ingv.it/. The catalogues of all relocated earthquakes and focal mechanisms parameters published in this study are available in the Supplementary Data File.
