# Supplementary material for: New seismological data from the Calabrian arc reveal arc-orthogonal extension across the subduction zone
Source: Sci Rep. 2021 Jan 12;11:473. doi: 10.1038/s41598-020-79719-8 (PMC7804144; doi:10.1038/s41598-020-79719-8)
Supplement: Supplementary file 5 — Supplementary Information 5. [file 41598_2020_79719_MOESM5_ESM.pdf]

New seismological data from the Calabrian Arc reveal arc-orthogonal extension across the subduction zone

Tiziana Sgroi[1,*], Alina Polonia [2,+], Graziella Barberi [3,+], Andrea Billi[4,+] and Luca Gasperini[2,+]

1 Istituto Nazionale di Geofisica e Vulcanologia, Sezione Roma 2, Rome, Italy

2 Istituto di Scienze Marine (ISMAR), CNR, Bologna, Italy

3 Istituto Nazionale di Geofisica e Vulcanologia (INGV), Osservatorio Etneo, Catania, Italy

4 Consiglio Nazionale delle Ricerche, IGAG, Rome, Italy

*corresponding author: tiziana.sgroi@ingv.it

+these authors contributed equally to this work

Supplementary data Figures and Datasets

Supplementary data Figures

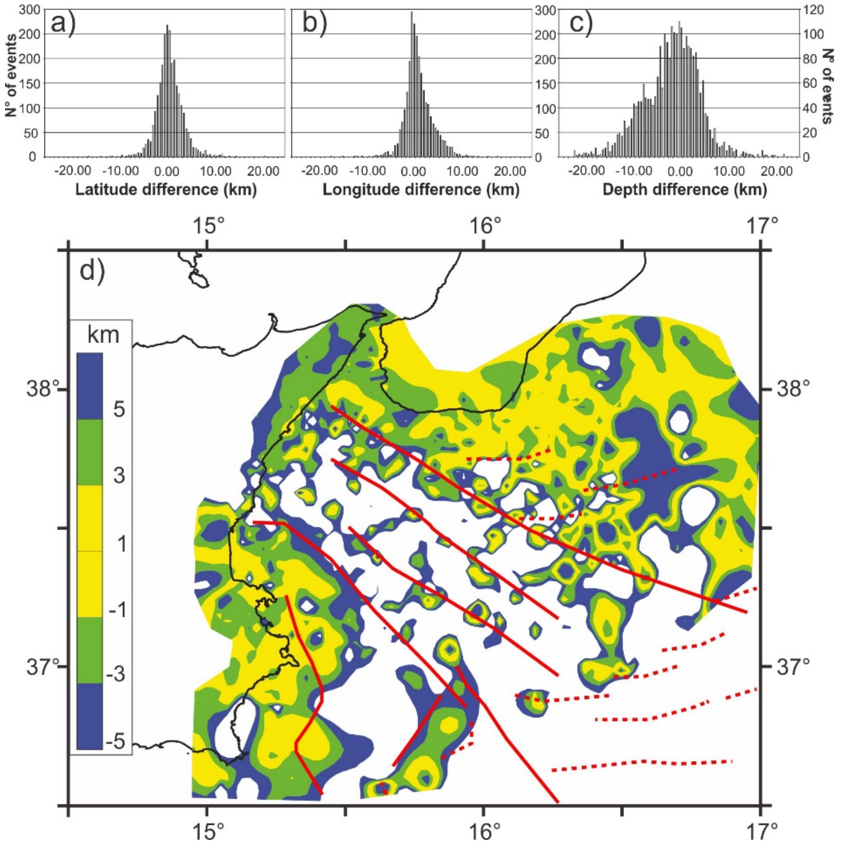

Figure S1: Statistical analysis performed on the difference between relocations from 3D and 1D models on latitude (a), longitude (b), and depth values (c). (d) Map of depth differences between earthquake relocations from 1D and 3D models. White areas highlight differences larger than ±5 km range. Main geological features including Malta escarpment, Alfeo–Etna Fault and Ionian Fault systems are sketched in red. The map was created using the software Surfer (Version 8.09.2391; http://www.goldensoftware.com/products/surfer). The plot was edited using Corel Draw 2018 (Version 20.0.0.633; http://www.corel.com).

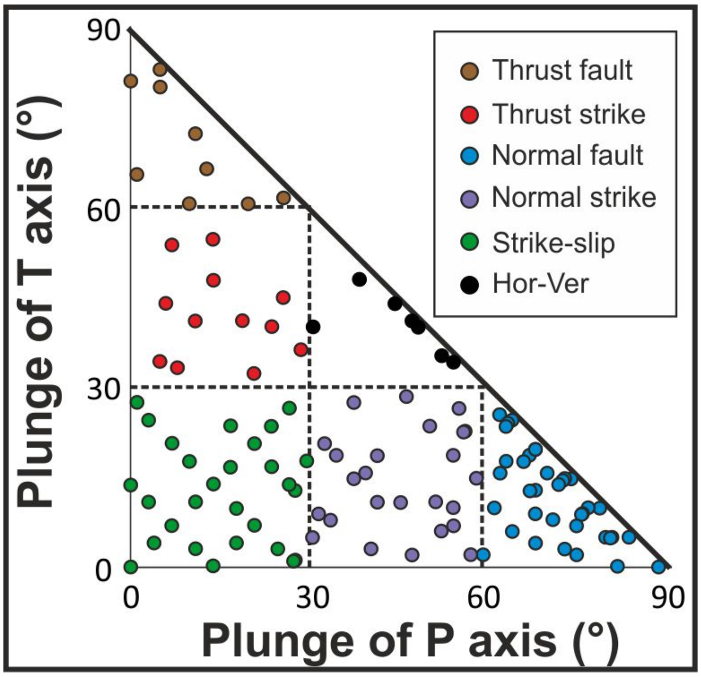

Figure S2: Classification scheme[1] based on the plunge of T-, B- and P-axes. The solutions are subdivided into six kinematic categories (thrust, thrust-strike, strike, normal, normal-strike, horizontal-vertical) and are represented schematically in a ternary diagram.

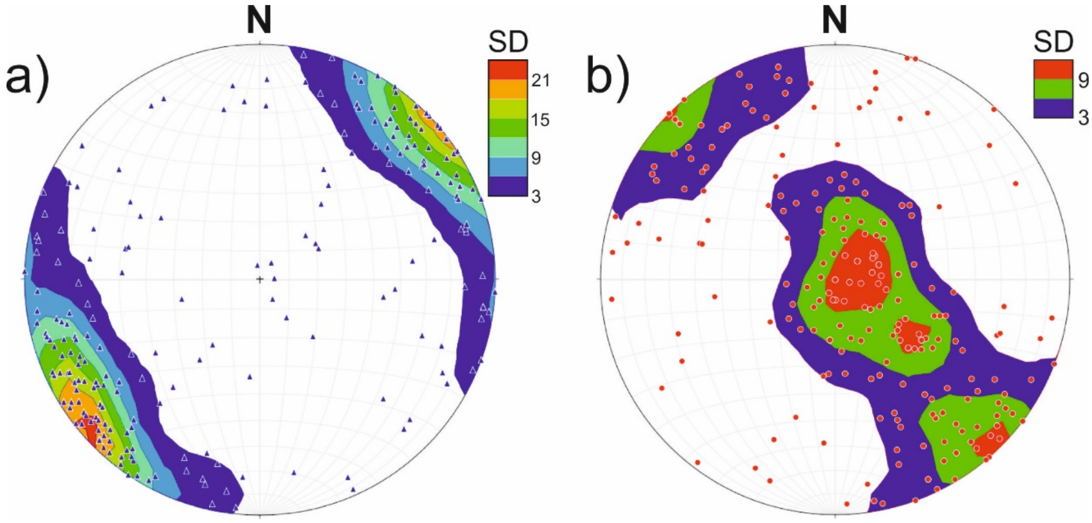

Figure S3: Density contours of T-axes (a) and P-axes (b) computed using the Kamb's method[2] from the earthquake focal mechanisms elaborated in this paper. Axes are projected in an equal-area polar plot. The figure was created using the software FaultKin (Version 8.1.2; http://www.geo.cornell.edu/geology/faculty/RWA/programs/faultkin.html).

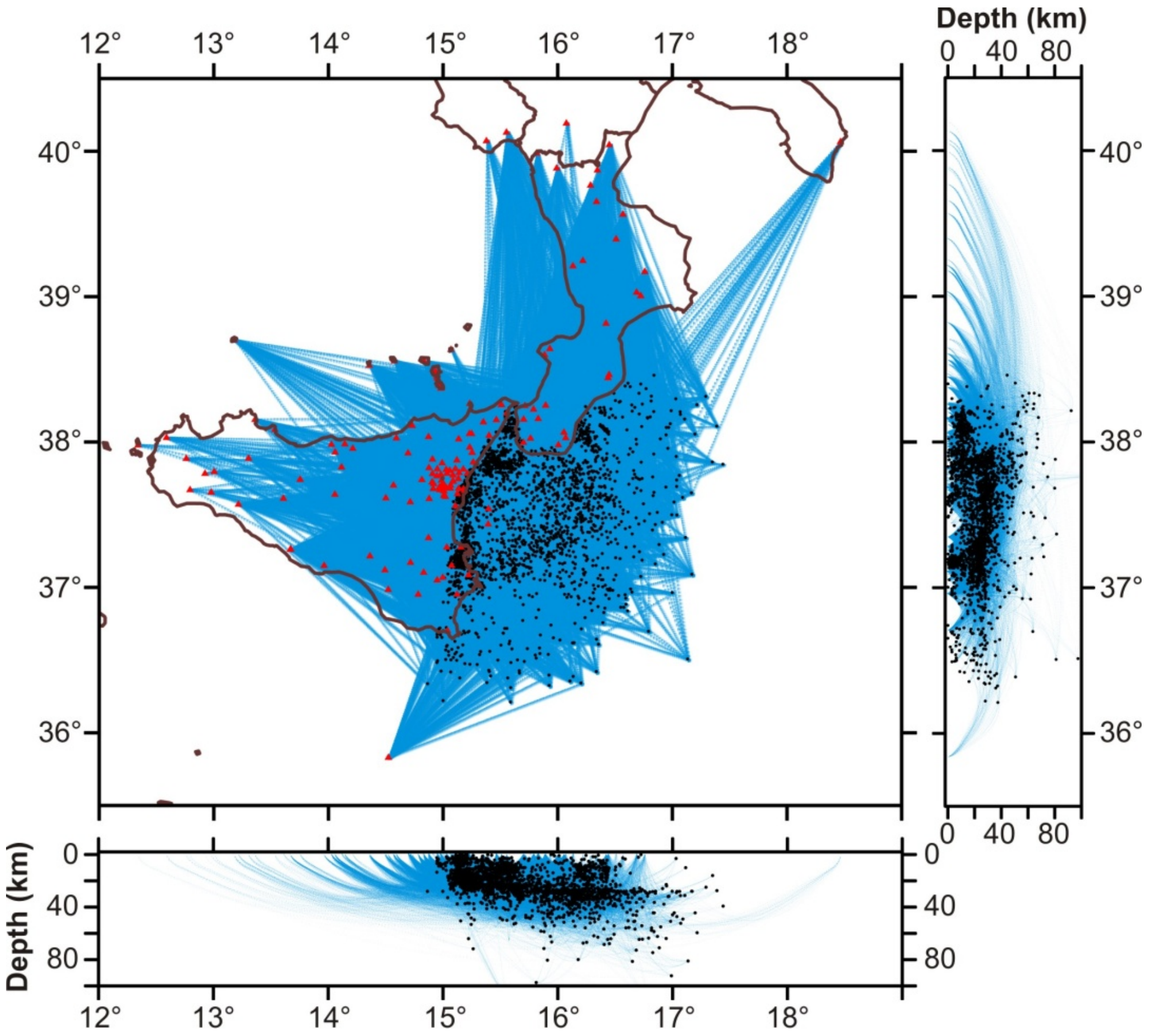

Figure S4: Map and W-E and N-S cross sections of P wave ray tracing used for the inversion of the 24256 P readings from the selected seismic events (black points), recorded during the 1990-2018 time span by both NEMO-SN1 and the permanent seismic stations (red triangles). The figure was created using the software Surfer (Version 8.09.2391; http://www.goldensoftware.com/products/surfer). The plot was edited using Corel Draw 2018 (Version 20.0.0.633; http://www.corel.com).

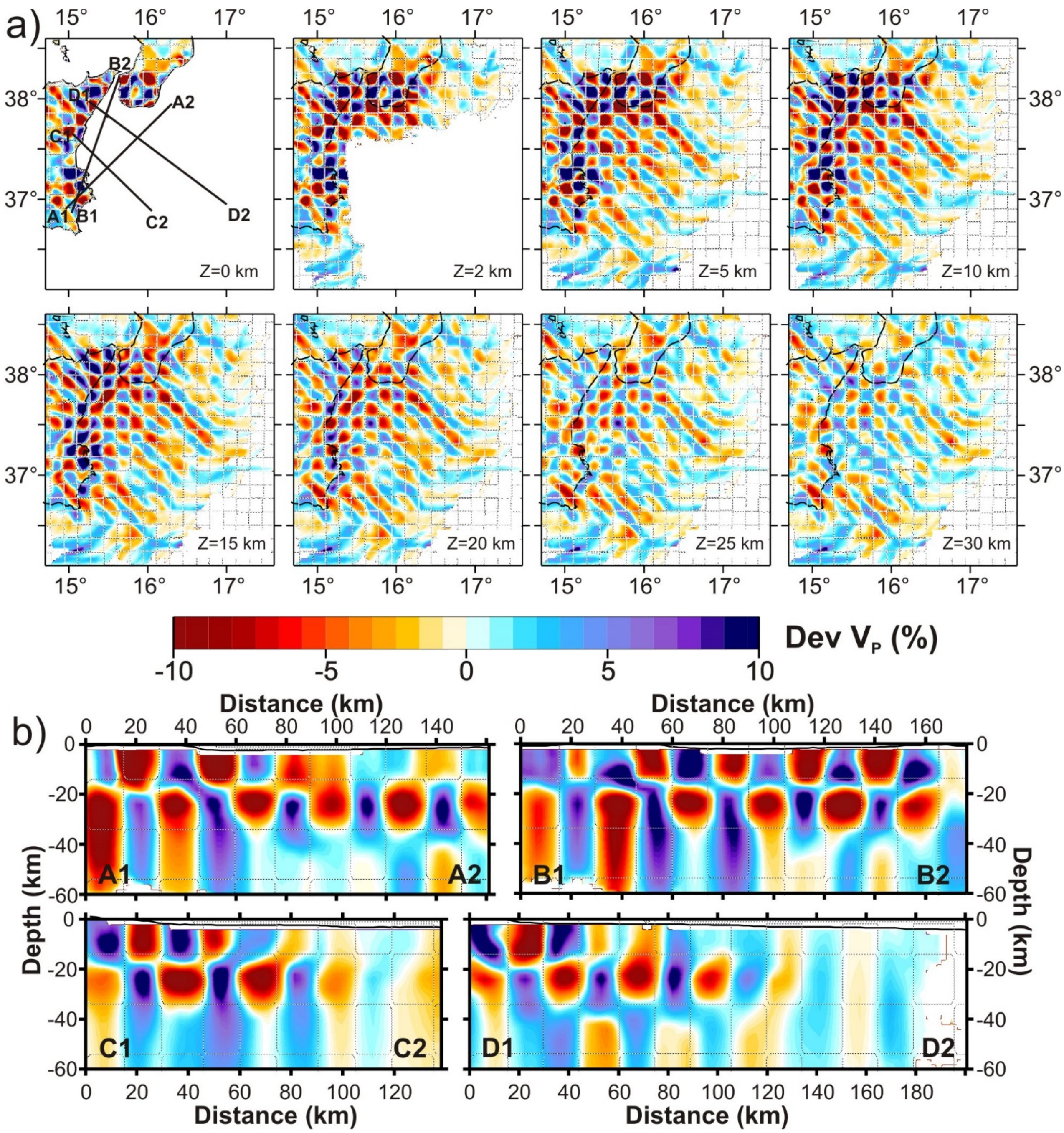

Figure S5: (a) Checkerboard synthetic test performed on the selected layers of the V_P model shown in Figure 6 having anomalies with lateral size of 15 km and amplitude ±10%. (b). Results of the checkboard test along the vertical sections with anomalies 15x20 km and amplitude ±10%. Grey thin lines in (a) and (b) highlight the configuration of the synthetic anomalies. The figure was created using the software Surfer (Version 8.09.2391; http://www.goldensoftware.com/products/surfer). The figure was edited using Corel Draw 2018 (Version 20.0.0.633; http://www.corel.com).

Datasets (Tables)

Table S1: Final relocations of 1020 earthquakes recorded both by land stations and the OBS hosted in the NEMO-SN1 seafloor observatory. The epicenters of 1020 final located earthquakes are shown in Figure 2b. Locations are computed using the Hypoellipse code[3]. See text for further details.

Table S2: 1D locations of 2658 crustal and subcrustal earthquakes (depth ranging 0-80 km) recorded in the period 1990-2018 by RSN and ERN. This dataset encompasses 138 events located with travel times recorded by NEMO-SN1 between October 2002-February 2003 and June 2012-May 2013. While travel times of the 138 events relocated with the use of NEMO-SN1 were handpicked, the travel times of the 2520 earthquakes were extracted from the ISB catalogue[4] and integrated with the catalogue of INGV – Catania[5]. To standardize the dataset, this catalog was further relocated using the new 1D velocity model and following the same procedure previously described. Map and W-E and N-S sections of the 1D relocated earthquakes (2658 events) are shown in Figure 3a.

Table S3: 3D locations of 2658 earthquakes recorded in the period 1990-2018. To test goodness and limits of the new 1D velocity model, the entire dataset, previously located using the new 1D velocity model, was relocated using a 3D velocity model computed for the Mediterranean[6]. Map and W-E and N-S sections of 3D locations are shown in Figure 3b.

Table S4: Focal mechanisms parameters computed on 223 earthquakes having a minimum number of eight clear polarities. Asterisk in the column related to the number of polarity indicates the presence of polarity from NEMO-SN1 in the focal mechanisms computation. The Cat column indicates the type of mechanism[1] (key: TF=thrust fault; TS=thrust-strike fault; NF=normal fault; NS=normal-strike fault; SS=strike slip; HV=horizontal-vertical). See text for further details.

References

1. Frohlich, C. Triangle diagrams: ternary graphs to display similarity and diversity of earthquake focal mechanisms. *Phys. Earth Planet. Inter.*, 75, 193–198 (1992).
2. Kamb, W.B. Ice Petrofabric Observations from Blue Glacier, Washington, in relation to theory and experiment. *J. Geophys. Res.,* 64(11) (1959).
3. Lahr, J.C. HYPOELLIPSE/version 2.0: a computer program for determining local earthquake hypocentral parameters, magnitude, and first motion pattern. *Open-File Report - U. S. Geological Survey,* 95, 89–116 (1989).
4. ISIDe Working Group. (2007). Italian Seismological Instrumental and Parametric Database (ISIDe). Istituto Nazionale di Geofisica e Vulcanologia (INGV). https://doi.org/10.13127/ISIDE.
5. Gruppo Analisi Dati Sismici (2019). Catalogo dei terremoti della Sicilia Orientale – Calabria Meridionale (1999–2019). INGV, Catania, http://sismoweb.ct.ingv.it/maps/eq_maps/sicily/catalogue.php.
6. Scarfì, L. *et al.* Slab narrowing in the Central Mediterranean: the Calabro-Ionian subduction zone as imaged by high resolution seismic tomography. *Scientific Reports*, 8:5178, https://doi.org/10.1038/s41598-018-23543-8 (2018).
